# Project-based learning approach to increase medical student empathy

**DOI:** 10.1080/10872981.2020.1742965

**Published:** 2020-03-20

**Authors:** Kyong-Jee Kim

**Affiliations:** Department of Medical Education, Dongguk University School of Medicine, Goyang-si, Korea

**Keywords:** Teaching and learning methods, empathy, project-based learning, medical student, Interpersonal Reactivity Index

## Abstract

This study investigated the impact of empathy training on medical students using a project-based learning approach on the development of their empathic abilities. Study participants were Year 2 students in the six-year medical program, to whom a one credit-hour course on empathic communication was offered over a semester. In this course, students worked on collaborative team projects in which they were asked to interview a person and to report the empathy that they shared with the interviewee. Development in student empathy was measured using the Interpersonal Reactivity Index inventory in a pre- and post-test format over the semester and their reflective reports were qualitatively analyzed to identify emerging themes on the lessons they learned from the project experience. A total of 45 students completed the questionnaires (a 97.8% response rate). Students’ overall empathy scores did not change over time (t = 1.943, p = .06). Still, students with lower empathy in the pre-test improved significantly in their empathy scores (t = 3.44, p < .01). Students reported that the empathy project was beneficial in improving their understanding of empathy and enhancing their empathic communication skills. This study shows the project-based learning approach was effective in developing medical student empathy especially for those with lower empathy.

## Introduction

Empathy is ‘a personality trait that enables one to identify with another’s situation, thoughts, or condition by placing oneself in their situation [1].’ Empathy is a key competence for the future workforce, so it has been emphasized in the curriculum in higher education. Moreover, empathy has been recognized as a key competence for healthcare professionals to improve patient–doctor relationships and to enhance patient satisfaction and outcomes [2]. Therefore, empathy has an important place in basic medical education from student selection to curriculum development.

Past studies of empathy in medical education have focused on assessing medical student empathy skills and on the psychometric properties of such instruments [1]. Research has also shown that there are individual differences in empathy in medical students [3] and it tends to decline over time [4,5]. Despite the growing interest in empathy in medical education, the literature is lacking in how to teach it, especially for medical students in very early years. Several studies have shown the effectiveness of various educational interventions in developing medical student empathy [6]. Still, past studies of educational interventions for promoting empathy for medical students have focused on students in clinical years, and research is lacking on medical students in very early years.

A previous study by Hutchison [7] found project-based learning was an effective instructional strategy for teaching empathy for college students in the interdisciplinary context. Project-based learning is an instructional model of inquiry-based learning and is rooted in constructivism which asserts knowledge and comprehension are constructed by the learner and are built upon his or her past experience [8]. Although research indicates project-based learning is effective in developing student empathy [7], little is known about using this instructional strategy to teach empathy to medical students. Thus, this study presents a project-based learning approach to develop empathy in entry-level medical students. This study investigated the effectiveness of the project-based learning approach in terms of changes in student empathy scores and their perceptions of this learning experience.

## Methods

### Study design and setting

An empathic communication course was designed and implemented in the six-year basic medical education program at Dongguk University, a mid-sized private medical school in South Korea, in 2019. This course was offered for Year 2 students in the pre-med phase in the curriculum, which aimed to develop students’ professional competencies required of tomorrow’s doctors, one of which was empathy. Empathy is a highly complex concept and it has not been clearly defined in medical education research [9]. In this course, a conceptual framework of empathy that encompasses both cognitive and emotional processes suggested by Howe [10] was adopted. As the students were in very early years in their medical study, this course did not explicitly touch upon clinical empathy, which takes place in the context of patient–doctor relationship [1]. Instead, this course intended to help students develop general competencies for empathy that laid the foundation for the development of clinical empathy, which is incorporated in the later phase in the curriculum.

The course was designed using a project-based learning approach to develop student empathy. It was expected that students need to apply the concept in the real-world context for them to develop empathic abilities and it was intended by adopting a project-based learning approach, they gain meaningful first-hand experiences in feeling empathy for others. This approach is also in line with the theory of first principles of instruction by Merrill [11] that learners need to integrate what they have learned in real-world settings. The project assignment in this course was to interview people who had backgrounds different from the students and were very active or passionate about their work. As the vast majority of students had just gotten out of high school, the project assignment was intended to give them an opportunity to communicate with people with backgrounds that they had not likely encountered with and to learn how to communicate with such individuals with empathy. By interviewing such individuals, it was expected that students would be able to understand their thoughts and feelings about their work from others’ perspectives.

Students were introduced to the project early on in the semester. Still, lectures were given throughout the semester on the concept of empathy and some communication skills, which are considered a key component of empathy [10]. As the theoretical foundation, students were introduced to the Satir model of communication styles [12] and an empathic communication skills model suggested by Pounds et al. [13]. Students practiced such communication skills through some small-group learning activities. The first half of the semester was spent in planning for the interview. Students worked in groups of 5–6 for the project assignment and each group organized an interview. Students contacted the potential interviewee by themselves and gained permission from him/her for the interviewee to take place. As the students had no prior experiences in interviewing people, it was considered that the interviewee needed to be readily approachable for them so they would not engage in communications too difficult for them to elicit the interviewee’s thoughts and feelings.

Each project group presented to the class why they chose the interviewee, what they would like to know about him/her and their interview plans including the list of interview questions. The interview questions included what the interviewees liked about their work and also the challenges or difficulties that they faced in their work so that students would understand the compassion and challenges in their work. Those who students interviewed included people with diverse backgrounds – *i.e*. a volunteer in the community about her experience helping those in need, a professor from overseas about living and working in Korea as a foreigner, and a kindergarten teacher about caring for little children. Each group recorded the interview in the video with the consent from the interviewee. Each interview took approximately one hour.

The second half of the semester was mostly spent in working on project deliverables. A successful project-based learning assignment concludes with a tangible artifact [14]. In this project assignment, students created a video as a final outcome of their project. Each group produced a video that included an introduction to their project (*i.e*. who the interviewee was and why they chose to interview him or her), some vignettes of the interviewee that they recorded during the interview, and the group’s reflection on the interview – what thoughts and feelings they shared with the interviewee and the meaning of empathy that they learned from their project assignments. The video was chosen as an artifact for this project because videos enable one to capture non-verbal signs the interviewee shows and to see whether students noticed them and responded effectively during the interview, which is an important component of empathic communication [13]. The groups were critiqued during the showcase of the videos in the final week of the course on how effective the empathic communication was, what lessons they learned about empathy from this project and the quality of their video production.

The course outcomes encompassed several competencies for tomorrow’s workforce to accomplish the project successfully in addition to enhancing empathic abilities. Students needed to be self-directed on their group work throughout the project. They needed to be creative and have good collaborative skills to produce a final artifact and work out issues raised during the process. Reflection was also a core component in this course, which is emphasized in promoting empathy [9]. Students worked in groups to review the interview they had recorded and to reflect on how effective their interviews were in terms of empathic communication. Moreover, students reflected their teamwork and what they learned about empathy from the project assignment by writing reflective papers as part of their final term papers.

As the project can be challenging for students, support and scaffolding were provided for them. Students received guidelines about what should be finished at what time in weekly class meetings. Students also received formative feedback throughout the semester by having groups to present the progress of their project during class and also through consultations with the instructor during project group meetings.

### Instrument and procedures

To evaluate the impact of student learning with the empathy project, students’ empathic abilities were assessed using the Interpersonal Reactivity Index (IRI) inventory developed by Davis [15]. The IRI is a self-report instrument for measuring empathy and its constructs encompass cognitive and emotional dimensions of empathy [15]. This 28-item instrument comprised four dimensions of empathy – *i.e*. perspective-taking, fantasy, empathic concern, and personal distress. This IRI has been reported valid and reliable in other studies [16,17] and has been widely used in medical education research [18]. In this study, the Korean version of IRI (K-IRI), which was translated and validated by Kang et al. [19] was used. This K-IRI has been used to assess empathy for Korean medical students and was found to have adequate psychometric properties [20].

Jefferson Scale of Physician Empathy (JSE) [21] is another instrument widely used to measure medical students’ empathy, and it is known as a valid and reliable tool [22]. JSE it was developed to measure empathy in the context of patient care and therefore assumes familiarity with medical practice [22,23]. Thus, it can be argued that JSE is not suitable for students in the very early phase in medical education without any clinical exposure yet. Accordingly, the IRI was chosen in this study, which was targeted to general populations [15]. Moreover, IRI captures both emotional and cognitive dimensions of empathy, whereas JSE regards empathy as primarily a cognitive process. As this course was built on the definition of empathy as both cognitive and emotional processes, the IRI seemed a better fit for the purpose of this study.

In this study, student empathy was measured in the first week of the course as a baseline and the measurement was repeated in the final week of the semester in a pre- and post-test format. Furthermore, qualitative data were obtained from students’ reflective papers and they were qualitatively analyzed to gain an understanding of student perceptions of how the project assignment influenced their knowledge, skills, and attitudes toward empathic communication. As research indicates various instruments for measuring empathy have different constructs, assessing empathy using one measurement tool is not valid [22]. Therefore, a qualitative research was also conducted in this study for a more in-depth understanding of the impact of student experiences with the empathy project on their development of empathy.

### Data analysis

Some descriptive and referential statistics were performed on the data obtained from the questionnaires. A paired Student *t*-test was performed to compare student empathy scores over time. An independent Student *t*-test was conducted to compare student empathy across genders. Students were divided into the lower empathy group and the higher empathy group based upon their pre-test empathy score using the median score. Changes in student empathy scores over time were compared between the groups using paired *t*-test. The data were analyzed using SPSS version 23 for Windows (IBM Corp., Armonk, USA), and statistical significance was accepted for *p* values <0.05.

To analyze the qualitative data, emerging themes or patterns were identified from the texts in all of the student reflective papers. The thematic analysis method [24] was used for data analysis. To establish the trustworthiness of the study, another investigator, a Ph.D. in education and experienced in qualitative research, audited the data analysis.

### Ethical consideration

This study was reviewed by the institutional review board of Dongguk University (DGU IRB 20,190,013). Consent was waived by the IRB because our data does not contain any individual person’s private or confidential information.

## Results

Forty-five students completed the questionnaires, a 97.8% response rate. Fifty-eight percent (n = 26) of the students were female and 42% (*n* = 19) were male. Student pre-test scores in empathy did not differ across gender (*t* = 1.30, *p* = .20). Student empathy scores were not compared across age groups, because they were relatively homogenous in terms of age, where the mean age was 20.1 (SD = 1.05). Cronbach’s alpha for the four sub-scales of empathy in the K-IRI ranged from .72 to .90, which demonstrated a high level of internal consistency.

### Changes in student empathy over time

Table 1 shows changes in student empathy over the semester. Students’ overall empathy scores did not change over time (*t* = 1.94, *p* = .06). Still, students with lower empathy in the pre-test improved significantly in their empathy scores over the semester (*t* = 3.44, *p* < .01). Empathy scores increased for students with lower empathy in three of four sub-scales in the empathy measurement; *i.e*. fantasy, empathic concern, and personal distress. Still, there was no change in empathy scores in the perspective-taking domain in the IRI for the lower empathy group (*t* = .64, *p* = .53). Student post-test scores in empathy did not differ across gender (*t* = .45, *p* = .64).

**Table 1.** Improvements in empathy scores over time for high- and low-empathy groups in the basement measure (Mean, SD)

Domains	Higher empathy group (n = 22)			Lower empathy group (n = 23)			Total (n = 45)		
	Pre-test	Post-test	t (*p*-value)	Pre-test	Post-test	t (*p*-value)	Pre-test	Post-test	t (*p*-value)
Perspective taking	3.54 (.39)	3.55 (.34)	.755 (.458)	3.36 (.36)	3.44 (.35)	.636 (.532)	3.54 (.39)	3.55 (.34)	0.165 (.870)
Fantasy	3.57 (.46)	3.68 (.36)	.369 (.717)	3.33 (.46)	3.56 (.25)	3.457 (.003)	3.57 (.46)	3.68 (.36)	1.883 (.068)
Empathic concern	3.58 (.50)	3.70 (.41)	1.637 (.120)	3.21 (.25)	3.54 (.34)	3.492 (.002)	3.58 (.50)	3.70 (.41)	1.727 (.092)
Personal distress	3.60 (.51)	3.71 (.38)	.718 (.483)	3.25 (.39)	3.55 (.35)	2.390 (.027)	3.60 (.51)	3.71 (.38)	1.623 (.113)
Total	3.57 (.38)	3.66 (.28)	1.538 (.142)	3.29 (.19)	3.51 (.22)	3.443 (.003)	3.57 (.38)	3.66 (.28)	1.943 (.060)

### Student reflections on the empathy project

#### Empathy as a novel experience and gaining new perspectives

Several students pointed out that this project offered them a novel experience of feeling empathy for others that they had first met. For instance, one student who interviewed a counselor wrote:

Through the process of preparing for and conducting the interview of her, I got to know more about her not only as a counselor but also as a mom, wife, and grad student. I got impressed with her endless passion for learning and got to understand the difficulties of her job as a counselor. I was surprised that I was able to have such an empathic feeling for someone whom I first met, who was not my family or longtime friends.

Another student also commented this project gave her a new experience of empathy:

Thankfully, our interviewee shared with us his life very openly and deeply, so I felt I was deeply immersed in the conversation with him. I never had such a heavy lingering experience about listening to someone else’s life story before. From this experience, I learned that empathy is more than just putting myself in someone else’s shoes but is to communicate with and care for him or her based on the in-depth understanding of the person’s life. So, I learned I had to try to fully understand the person’s life with empathy in order to have meaningful relationship with others.

Several students also reflected that such a novel experience in feeling empathy opened up new perspectives for them. One student who interviewed a college professor who teaches her students passionately stated:

I felt empathy for the professor who had lived a life very different from mine when she shared her life stories with us. She had a lifestyle of pursuing changes and seeking new things. I was not familiar with her lifestyle because it was very different from me, my family members, and people around me. I was even a kind of person sometimes who was afraid of changes. But, I got to understand her lifestyle from her perspectives after listening to her life experiences. This made me reconsider the lifestyle of mine. I learned from this experience that communicating with others with empathy helps me gain new perspectives.

#### Importance of empathy for building relationship with others

Many students pointed out that they learned the importance of empathy as a foundation for building good relationship with others. A student who interviewed a college professor in literature wrote:

I learned empathy is essential for building relationships with people from diverse backgrounds such as age, gender, or ethnicity. Being a medical student, I did not expect I would keep in touch with the professor who teaches literature and talk over many things even after her class was over. This experience motivated me to communicate with people with diverse backgrounds to get to know more about them. I learned the importance of empathy to communicate with people from diverse backgrounds.

Another student who interviewed a high school teacher also stressed the importance of empathy in understanding others’ perspectives as a basis for building up good relationship:

The meaning of empathy that I learned from this project is to understand other people’s perspectives. Everyone has different backgrounds, so people look at the same issue differently, however subtle or big the difference is. I think empathy is important for building good relationships, because without it people think from their own perspectives only. For instance, I used to look at the issue of restrictions on hair styles for high school students from the student’s perspective only. By interviewing the high school teacher in this project, I came to an understanding that students, teachers, and school administrators all have different perspectives on this issue. Without having empathy for others, they will have to argue endlessly with each other. So, I felt empathy is to acknowledge differences and try to understand other’s perspectives.

#### Enhanced empathic communication skills

Most of the students also stated that this project experience helped them learn effective empathic communication skills. One student who interviewed a leader of a college student organization commented:

I tried to practice empathic communication during the interview by showing empathy to him (the interviewee). I think this made the communication went well. He talked more openly in response to my reactions that I showed him. The conversation lingered longer to my memory when I listened to him with empathy. I learned the importance of empathy for effective communication from this experience. When the other person feels that I am listening to him or her with empathy, I can communicate with him or her better and I can share thoughts more deeply with that person. I think this makes positive influence for both of us because such a conversation is not easily forgotten and lasts in my mind much longer.

Another student who interviewed a counselor reflected on how his communication style has changed to a congruent one, which is regarded to be an ideal one suggested by the Satir model [12], by applying empathic communication skills:

My communication style used to be the super-rational one who thinks logically, rarely showing my emotions. I think I did not easily get close to other people because of that. So, I felt quite difficult about being empathic for others when the project started. But, as I worked on this project, my communication style became a more congruent one. I got to show more empathy in the language and gestures that I expressed as I interviewed her. I got to respond to her actively as I actively listened to her and I also got to have more questions for her. This kept the conversation going. I think my communication style has become more congruent between my thinking and expressions as I felt more empathy for her. I realized the importance of empathy from this experience. When I treat the person with sincere respect and show empathy for him/her, I can communicate better with that person and it helps me build up good relationship with him or her.

## Discussion

This study investigated changes in student empathy scores over time as an outcome of the project-based learning. Students' overall empathy scores did not improve over the semester, although there was an enhancement among those with lower empathy in the pre-course test. This finding indicates the impact of student learning may differ across students as there are individual differences in empathic abilities among them [3]. This study shows this project-based learning approach more likely benefits students with lower empathy in developing their empathic abilities.

Students’ reflections indicate that they had a novel experience of feeling empathy for others from the project assignment and that they gained new perspectives from such experiences. Students also indicated improvement in their empathic communication skills from this project in their reflective papers. It can be argued that this project-based learning approach was effective in developing students’ emphatic abilities by giving them an authentic and sometimes novel experience in communicating with people with diverse backgrounds and having them delve into others’ thoughts and feelings from others’ perspectives.

This study has a limitation in that changes in student empathy scores were measured for a relatively short period of time. Moreover, this study did not engage students in clinical empathy. Therefore, this study does not provide direct evidence into how students’ project-based learning experience in this study influences their development of clinical empathy. A longitudinal study of the impact of this project-based learning experience on the student development of clinical empathy is recommended for future research using measurements fit for the clinical context. More importantly, student experiences in this empathy project were limited as they interviewed people they might have perceived as positive role models – *i.e*. teachers, professors, student leaders, and counselors. People students had encountered in this project did not include those in difficult situations whom might be more challenging to communicate with. Future study is warranted to investigate the impact of student experience in engaging in more difficult communications on their empathy development, and how such experiences differed from those when they encountered positive role models. Still, it is expected students are better prepared to engage in difficult commutations with empathy by experiencing the empathic communication with positive role models in this project assignment. Furthermore, future study is warranted of the impact of project-based learning on the development of students’ collaboration skills and creativity, which were also expected to be learning outcomes of the project in this study.

## Conclusion

While the study did not show overall improvement in student empathy, the project-based approach had some impact on those with low empathy. Moreover, the empathy project was beneficial in improving students’ understanding of empathy and enhancing their empathic communication skills.
